# Needs Assessment for a Decision Aid in Oral Cancer Requiring Major Resection and Reconstructions

**DOI:** 10.1002/ohn.70071

**Published:** 2025-11-18

**Authors:** David Forner, Victoria Taylor, Martin Corsten, Valeria E. Rac, Sonia Meerai, Andrew G. Shuman, Sharon Tzelnick, Rosemary Martino, John R. de Almeida, David P. Goldstein

**Affiliations:** ^1^ Department of Otolaryngology–Head & Neck Surgery University of Michigan Ann Arbor Michigan USA; ^2^ Division of Otolaryngology–Head & Neck Surgery Dalhousie University Halifax Nova Scotia Canada; ^3^ Program for Health System and Technology Evaluation, Toronto General Hospital Research Institute (TGHRI) University Health Network Toronto Ontario Canada; ^4^ Ted Rogers Centre for Heart Research at Peter Munk Cardiac Centre, Toronto General Hospital Research Institute (TGHRI) University Health Network (UHN) Toronto Ontario Canada; ^5^ Institute of Health Policy, Management and Evaluation (IHPME), Dalla Lana School of Public Health University of Toronto Toronto Canada; ^6^ Faculty of Education and Health, School of Social Work Laurentian University Sudbury Ontario Canada; ^7^ Department of Otolaryngology–Head & Neck Surgery the University Health Network Toronto Canada; ^8^ Department of Speech‐Language Pathology University of Toronto Toronto Ontario Canada

**Keywords:** head and neck cancer, oral cavity cancer, quality of life, shared decision‐making

## Abstract

**Objective:**

Treatment of advanced oral cavity cancer necessitates ablative and reconstructive surgery that can be life‐altering, creating nuanced priorities between the desire for survival and quality of life. This study sought to describe currents practice of shared decision‐making among patients with advanced oral cavity cancer and determine the need for a decision support tool.

**Study Design:**

Cross‐sectional, convergent, mixed methods study from 2020 to 2023.

**Setting:**

Two major Canadian academic centers.

**Methods:**

Semi‐structured interviews were conducted and interpreted via inductive thematic analysis of preoperative and postoperative patients with locoregionally advanced oral cavity cancer. Qualitative findings were integrated with data obtained from validated instruments that examined shared decision‐making (SDM‐Q‐9), decisional conflict (Decisional Conflict Scale; DCS), and decision‐making self‐efficacy (Ottawa Decision Self‐Efficacy; ODSE).

**Results:**

The median age of the 37 participants was 67 years (SD: 11). Qualitative themes suggested that the following influenced care decisions: (1) approaches to information delivery, (2) preoperative experiences impact decision‐making, (3) perceived knowledge gaps and negative emotions, and (4) fear of cancer. Seven patients (18.9%) had clinically significant decisional conflict (DCS > 25) despite high levels of decisional self‐efficacy (mean ODSE 95.1, SD:7.9). Greater perception of shared decision‐making (*r* = −0.328, *P* = .048) and decisional self‐efficacy (*r* = −0.687, *P* < .001) were correlated with lower decisional conflict. Recommendations for future decision‐making tools included: (1) wide accessibility, (2) timeline of treatment events, (3) incorporate components that activate shared decision‐making and integrate the clinician‐patient dyad, and (4) promote conversation around difficult topics.

**Conclusion:**

These findings support the need for integrated and improved tools to promote shared decision‐making.

Advanced oral cavity cancer results in significant alterations to breathing, speech, swallowing, and appearances. Surgical resection and reconstruction is the primary treatment for these cancers, but disease and treatment‐associated complications can be substantial. Many patients with head and neck cancer are older and frequently have significant comorbidities related to age and cancer‐associated risk factors.^1^ These factors can create nuanced priorities between the desire for survival and quality of life.^2^

Despite advances in surgical technique and adjuvant treatment, the potential for poor oncological and functional outcomes remains. In this vulnerable patient population, patient centered care through shared decision‐making is crucial and there is a growing body of evidence that unmet information needs may contribute significantly to psychological distress.^3^

Owing to the burdens of both the disease and its treatment, the shared decision‐making process is particularly important to determine how patients grapple with the decision to have surgery for head and neck cancer. While the potential for cure may be a clear choice for some, others may have preference for less invasive noncurative intent treatment. This area is understudied in the head and neck cancer population and there is a paucity of evidence regarding patient and surgeon experiences.

Decision aids improve the shared decision‐making process and reduce decisional conflict, bolster knowledge, and improve value‐choice congruence.^4^ To date, no decision aids for oral cavity cancer have been developed.

We therefore aim to answer the questions: To what degree do patients take part in the decision‐making process, do they experience decisional conflict, and is there a need for development and implementation of a decision aid for adult patients considering major surgery for advanced oral cavity cancer?

## Methods

This protocol was prepared in accordance with elements of the Strengthening the Reporting of Observational Studies in Epidemiology (STROBE) and Standard for Reporting Qualitative Research (SRQR) guidelines,^5^ as well as with consideration of best practices in mixed‐methods health services research.^6^, ^7^, ^8^

### Study Design

This study was a cross‐sectional, convergent, parallel mixed‐methods design at 2 major academic centers in Canada (Queen Elizabeth II Health Sciences Center, Halifax, Nova Scotia, and the University Health Network, Toronto, Ontario) from February 2020 to March 2023. This study was approved by both the University Health Network and Nova Scotia Health Authority institutional review boards.

### Study Population

Adult (>50 years old) patients with oral cavity cancer considering surgical ablation and regional or free flap reconstruction were eligible for the study. Patients from both the preoperative and postoperative periods were recruited. Preoperative patients remained eligible if they elected to not undergo surgery. Postoperative patients did not have to participate in the study during their preoperative phase.

Exclusion criteria included: patients with distant metastatic or unresectable disease, medical comorbidities obviating surgery with curative intent, and previous head and neck cancer requiring surgical ablation and regional or free flap reconstruction.

Participants were recruited until theory saturation had been reached, defined as no generation of new themes across research sites.^9^

Purposive sampling was employed as per standard practices in qualitative research.^10^ Specifically, maximum variation purposive sampling was utilized to ensure important shared patterns and themes emerged from a purposefully heterogeneous sample representative of the oral cavity cancer population. Participants were recruited to capture age, gender, comorbidity burden, and the preoperative and postoperative periods, including both the early and late postoperative periods.

### Objectives

The primary objectives of the study were to determine current patient‐perceived extent of involvement in the shared decision‐making process, and to determine the need for a decision aid during surgical consultation for advanced oral cavity cancer, including exploring the level of decisional self‐efficacy and decisional conflict experienced by patients.

The secondary objective of this study was to determine content and route of administration for potential decision aids in this setting.

### Quantitative Data and Analysis

The quantitative methods included both demographic information collection as well as a series of health measurement instruments dedicated to determining patient perceptions of shared decision‐making involvement, decisional conflict, and decisional self‐efficacy. Collected demographic information included age, sex, education level, income, ethnicity, medical comorbidities (operationalized as the age‐adjusted Charlson Comorbidity Index^11^), history of previous head and neck cancer, history of previous oral cavity resection and/or reconstruction, and self‐identified communication issues.

#### Shared Decision‐Making

The Nine‐Item Shared Decision‐Making Questionnaire (SDM‐Q‐9) is a well‐established and validated instrument that measures the patient's perspective in the shared decision‐making process.^12^, ^13^ The scale has been validated in oncologic research settings and consists of 9 items rated on a 6‐point scale from “completely disagree” (scored 0 points) to “completely agree” (scored 5 points). The raw score is transformed, resulting in a range from 0 to 100. On this scale, 0 represents the lowest possible perception of involvement in the shared decision‐making process and 100 represents the highest perception.^14^

#### Self‐Efficacy

The Ottawa Decision Self‐Efficacy Scale (ODSE) allows patients to reflect on how confident they feel in making an informed choice about their medical care.^15^ Self‐efficacy is the self‐confidence or belief a patient has in their ability to obtain and act on decision‐making information, and the support of self‐efficacy is essential for patient involvement in the shared decision‐making process.^16^ The ODSE is an 11‐item questionnaire that has been validated in multiple populations and accurately measures patient self‐efficacy.^16^, ^17^, ^18^

#### Decisional Conflict

The Decisional Conflict Scale (DCS) is a 16‐item questionnaire with 5 response categories from “strongly agree” (scored 1 point) to “strongly disagree” (scored 4 points). Transformed final scores above 25 are considered to be clinically relevant conflict.^19^

### Qualitative Data and Analysis

Semi‐structured interviews were performed by a single research assistant (VT). Interviews were conducted over the telephone, recorded, and transcribed verbatim by an experienced medical transcriptionist. Participant interviews took place at least 1 week after their initial consultation with head and neck surgical oncology.

The semi‐structured interview (Supplemental Figure S1, available online) consisted of questions intended to elicit patient experiences in the shared decision‐making process and their thoughts on the utility of a decision aid. The interview script was pilot tested on 4 participants to ensure it facilitated discussion on the intended subjects.^20^

Qualitative analysis was performed through an inductive thematic analysis approach (SM, VER) using the Braun and Clarke framework.^21^ Qualitative analysis results were further discussed with a junior and senior head and neck surgeon (DF, DPG) and contextually refined.

### Mixed‐Methods

Integration of data pertaining to the primary objectives was accomplished at the interpretation level via side‐by‐side joint display.^22^, ^23^ Joint displays assist the data integration process in mixed‐methods research and took the form of an integrated results matrix in this study. Joint displays are a common way to “mix” data in mixed‐methods studies and were chosen for this study for improved visualization of the data.^24^

## Results

### Participants

There were 37 participants in the study, all of which completed the ODSE, SDM‐Q‐9, and DCS. Nine surgeons took participated in the study across the 2 participating centers. Thirty‐one participants took part in semi‐structured interviews. Three patients were missing demographic data. The median age of participants was 67 years (SD: 11) and 55% were male. Most patients had a substantial comorbidity burden (average CCI:5). For patients in the postoperative period (n = 10), the average time between surgery and interview was 259 days (range 49‐531 days). Sociodemographic data from one participating center was available, wherein all patients were White (100%), the majority completed high school or less (73%), and most had annual income of less than $60,000 CDN (63%).

### Primary Objectives—Shared Decision‐Making and Decision Aid Needs Assessment

Qualitative themes included: (1) approaches to information delivery, (2) preoperative experiences impact decision‐making, (3) knowledge gaps and negative emotions, and (4) fear of cancer. Supporting quotations are found in Table 1.

**Table 1:** Supporting Quotations for Identified Themes

Theme	Supporting Quotations
Approaches to Information Delivery	*“Well, just exactly what they had to do, what the process was going to be – remove the teeth, shave the jaw, just all the process and the doctor actually drew it on the board and showed me what had to be done.”*
	*“…you have to have a doctor that is sympathetic to the emotional concerns the patient has, that explains what the problem is carefully and simply so there is no misunderstanding. And, I think it has to begin as promptly … so that it doesn't drag on.”*
	*“I just wanted the data, the information, the challenges and you know, I realize the situation I am in but it is a business decision and you make it based on the best data available and you take the risk into consideration, and you make what is believed to be the best choice by exploring many options and weighing the pros and cons.”*
Knowledge Gaps and Negative Emotions	*“I just didn't know what to expect. Would I be able to talk with that in … how long does it stay in and just what – I have never had one and so I didn't know what to expect. Just give me some idea… how does it affect you?”*
	*“What you did and what you told me you were going to do were miles apart – what did you do – go from Plan A to Plan B? …He said no we did exactly what we intended to do. And I thought well, that ain't what you told me!”*
Pre‐Operative Experiences Impact Decision‐Making	*“…and I feel funny in my mouth of course… and down the road I don't know, let's say there may be dentures so that I can chew. You know what I mean? It is very important for me that I am able to chew.”*
Fear of Cancer Impacts the Decision‐Making Process	*“Well, it is kind a little bit scary and I was thinking about that on my way back home and it is a bit scary because I know it is a big operation … and so it makes you kind of wonder, you know?”*
	*“You know, it seems to me it was do or die. It wasn't a preference kind of thing.”*
	*“It is about surviving. You have to know what comes ahead of you and how you deal with it afterwards.”*

#### Approaches to Information Delivery

Participants overall felt included in the decision‐making conversation and discussed weighing the pros and cons of surgery with their surgeon, including future risks. Patients valued a pragmatic approach, not an emotional one, to delivering the information regarding surgery, noting that it was discussed in a formalized fashion, beginning with fact gathering, taking risks into consideration, exploring options, and finally coming to a decision. Participants felt it was a shared decision to pursue surgery, and that their surgeon was thorough in communicating all information around treatment with them. This was true of both preoperative and postoperative participants.

#### Preoperative Experiences Impact Decision‐Making

Patient experiences during the preoperative setting impacted decision‐making regarding both surgery and postoperative care. Outcomes of this process also shifted participant priorities in their own life, which is a key consideration for shared decision‐making as it allows patients to identify and articulate their own values.

Information shared by the surgeon increased trust and confidence between the patient and clinician. Participants commonly expressed their concerns for the recovery period, which was subsequently explained by surgeons further decreasing participant stress levels. Patients described feeling uncertainty and acceptance of that uncertainty, as well as receiving extensive information and then undergoing periods of waiting for the next step in their care.

#### Knowledge Gaps and Negative Emotions

There were gaps in knowledge specific to the nature of the surgery, resulting in uneasiness and increased stress experienced by patients. For example, there were feelings of apprehension around the healing and recovery process, specifically around expected timelines—participants placed value on having more information about these expectations. Uncertainty about what to expect with a tracheostomy tube, postoperative aesthetic outcomes, and the need for adjuvant radiotherapy were common issues brought forward by participants. Medical jargon was a frequent barrier for patients that did not have extensive knowledge of these definitions and how they related to their current health and upcoming surgery.

#### Fear of Cancer Impacts the Decision‐Making Process

Patients articulated their overall fear of cancer, including the life‐threatening nature of cancer, its impact on the decision‐making process, and sense of urgency in seeking treatment. Many patients expressed they believed there was no decision to be made, identifying that survival was the highest priority.

Patients described overall high levels of perceived shared decision‐making, with mean SDM‐Q‐9 scores of 86.3 (SD: 18.3). The lowest score was 42. There was a minimal ceiling effect, as 15 (40%) of participants reported the maximum possible score (100).

The mean DCS score was 10.5 (SD: 13.3). Seven patients (18.9%) had clinically significant decisional conflict (DCS > 25) despite high levels of decisional self‐efficacy (mean ODSE 95.1, SD: 7.9). Greater perception of shared decision‐making (*r* = −0.328, *P* = .048) and decisional self‐efficacy (*r* = −0.687, *P* < .001) were correlated with lower decisional conflict. In an exploratory post‐hoc analysis, there was no correlation between age or comorbidity burden with SDM‐Q‐9, DCS, or ODSE.

### Secondary Objectives—Contents of a Decision Aid

Overall, participants were supportive of the creation and utilization of a decision aid in this setting. Several recommendations for future decision‐making tools also arose during the interviews, including: (1) wide accessibility, (2) timeline of treatment events, (3) incorporate components that activate shared decision‐making and integrate the clinician‐patient dyad, and (4) promote conversation around difficult topics.

#### Wide Accessibility

Patients identified multiple formats that information could be presented including printed materials and online including audio and visual integration. Other considerations for accessibility include viewing the patient holistically—meaning that patients may be navigating and juggling multiple life responsibilities, financial responsibilities, and other health issues.

#### Timeline of Treatment Events

A visual timeline of events provides patients with an easier entry point to reflect on what they are required to do for their appointments, leading up to surgery, and in the recovery period. According to some participants, valuable information to include in the timeline includes details on overnight accommodation, what to do after waking up from surgery, and overall procedure and recovery information.

#### Activate Shared Decision‐Making

Patients recommend that future decision aids integrate the advantages and disadvantages to surgery, demonstrations of surgical options, and patient values and preferences. The decision aid should include surgical and nonsurgical options, risks to those options including recovery, sensation, scarring and functionality, and expected result of not pursuing surgery. Additionally, participants think it would be meaningful to hear about the details and experiences from someone who has undergone similar treatment. Finally, it was recommended that the decision aid assist in integrating other parties into the decision‐making process, such as family members.

#### Promote Conversation Around Difficult Topics

Patients also highlighted that decision aids should help individuals have discussions around the difficult topics that emerge around their diagnosis and treatment with the people in their life (palliative and end of life care, short‐ and long‐term daily living supports). Importantly, mental health assessment and access to support embedded in the decision‐making tool would address overall patient burden and fear of their cancer diagnosis and next steps for care.

### Integration

We created a joint display to mix our quantitative and qualitative methods (Table 2). Overall, the data exhibited confirmation. Shared decision‐making perceptions were high from patients, and participants felt included in the decision‐making conversations. In terms of the needs assessment for a decision aid, while most participants had low decisional conflict, nearly one‐fifth still reported clinically significant conflict. Both shared decision‐making and decision self‐efficacy lowered decisional conflict, and a decision aid could improve both those factors. This aligned with qualitative reports of patients valuing pragmatic approaches to information delivery, and reducing knowledge gaps resulting in lower distress. While participants felt a decision aid would be helpful, many patients expressed they believed there was no decision to be made, demonstrating subtle discordance.

**Table 2:** Joint Display for Integration of Mixed Methodologies

Objective	Quantitative result	Qualitative support
Shared Decision‐Making	1. Mean SDM‐Q‐9: 86.3	1. Participants overall felt included in the decision‐making conversation
		2. Participants felt it was a shared decision to pursue surgery
Decision Aid Need	1. 19% of patients experience significant decisional conflict	1. Patients valued a pragmatic approach to information delivery
	2. SDM and decisional self‐efficacy associated with lower decision conflict	2. There were gaps in knowledge specific to the medical process of the surgery, resulting in uneasiness and increased stress experienced by patients
		3. Information shared by the surgeon increased trust and confidence between the patient and clinician
		4. Many patients expressed they believed there was no decision to be made, identifying that survival was the highest priority^a^

Abbreviation: SDM, shared decision‐making.

^a^ Discordant results between quantitative and qualitative.

## Discussion

In this multi‐institutional mixed‐methods study we have characterized patient perceptions of shared decision‐making, decision self‐efficacy, and decisional conflict for the first time in patients with locally advanced oral cavity squamous cell carcinoma. This cohort, consisting of patients from 2 major academic centers in Canada, showcases patients generally had a positive attitude toward their participation in the decision‐making process. Despite both high levels of shared decision‐making and decision self‐efficacy, decisional conflict remained high in this group, likely reflecting the differences in patient values and preferences from expected functional and oncologic outcomes. Patients have confirmed the need for a decision aid in this setting.

There is a growing body of literature investigating shared decision‐making, decisional conflict, and decision regret in patients with head and neck cancer. The majority of this literature focuses either on heterogenous groups of head and neck cancer patients, or on scenarios where clinical equipoise exists, such as in HPV‐mediated oropharyngeal cancer or early‐stage laryngeal cancer. Locoregionally advanced oral cavity cancer presents a different scenario whereby clinical equipoise is not present, but instead patient preferences and values may differ from the singular best medical treatment in the form of surgical resection and reconstruction. Specifically, while some patients may favor invasive, curative intent treatment, others may have values and preferences that do not align with curative intent surgery.

In a recent study by Hosseini and colleagues, decisional conflict was assessed 2 weeks after initial consultation in a heterogenous group of head and neck cancer patients.^25^ Only 43% felt the decision to undergo treatment had been shared with their physician, and almost half of patients experienced decisional conflict. While the decisional conflict experienced in our cohort was lower, there were similar themes identified in both studies, including patients being uncertain of options available to them and what the risks, benefits, and post‐operative effects would be. Similarly, a recent systematic review of patients with head and neck cancer showed clinically significant decisional conflict was present in 22% to 47% of patients and up to 70% of patients experienced decision regret.^26^ Similar themes were also found among our patients, with reception of complicated information and lack of practical information being common. While not assessed quantitatively in our study, decision regret among head and neck cancer patients was likewise analogous, with patients being affected by altered appearance, functional consequences, and ambivalence about their diagnosis.^26^ Windon and colleagues showed similar complex findings among their patients as were highlighted in our study.^27^ Patients felt both highly deferential in the decision‐making process to their physician but ultimately endorsed the presence of shared decision‐making.

Integration of data within this mixed‐methods study overall resulted in confirmation between the quantitative and qualitative aspects. There was subtle discordance in which some patients discussed that there was no decision to be made but overall reported high levels of perceived shared decision‐making. This may be due to an overall lack of understanding that a supportive care approach is still an active treatment choice. Many patients felt survival was the only decision to be made and quality of life was not necessarily factored into this decision. Interestingly, this is at odds with other studies within the head and neck cancer literature, where quality of life frequently trumps survival.^28^ Facilitating discussion on whether curative intent therapy aligns with patient preferences is one such benefit to a decision aid when clinical equipoise does not exist.

While patients in our study reported high perceptions of shared decision‐making, clinically significant decisional conflict was still experienced, and patients felt that a decision aid would be beneficial. Participants provided concepts that should be incorporated into such a decision aid, including wide accessibility, providing a timeline of events, activating the shared decision‐making process, and promoting difficult conversations. Promotion of shared decision‐making, including elucidating patient values and preferences, is needed. Among laryngectomy patients, Raol and colleagues demonstrated that physicians documented patient fears and beliefs regarding quality of life for only 27% of patients.^29^ Similarly, we have previously shown that advance care directives are only documented in 10% of patients undergoing surgery for head and neck cancer.^30^ As highlighted by the participants in the current study, promotion of end‐of‐life wishes through a decision aid may be of benefit. As evidenced by a reduction in decisional conflict with improved shared decision‐making and decisional self‐efficacy scores, a decision aid may reduce patients' conflict and distress. While participants of this study did generate thoughtful ideas for a future decision aid, formalized decision aid creation methodology must be followed.^31^

The findings of this study must be contextualized. While post‐operative patients were recruited from a wide range of intervals from their surgical date, the median time since surgery was relatively long and may have impacted what participants felt was important during the decision making process. On the other hand, this may have allowed for more accurate reflection of their personal values. Recruitment during this study was prolonged due to the COVID‐19 pandemic and its effects on both clinical care and research endeavors. Furthermore, participants would have been less likely to be out in public during the pandemic and therefore may have placed less emphasis on functional outcomes in terms of social interaction and cosmesis. We did not specifically investigate if all patients received discussion surrounding the potential need for adjuvant therapy, which may have been an influential factor in their decision to pursue curative intent treatment at all. Lastly, while this study was not designed to assess the interaction of specific sociodemographic factors on the decision‐making process, such factors may be expected to have an inherent association. We provide these demographic factors from a single institution. Future work should more closely investigate how these factors interplay with the decision to undergo major ablative and reconstructive surgery.

## Conclusion

Patients with locally advanced oral cavity squamous cell carcinoma positively portrayed their involvement in the shared decision‐making process but endorsed the potential benefit of a decision aid. A significant proportion of patients experience decisional conflict that may benefit from a decision‐making tool to enhance their involvement in their own care and to facilitate necessary conversation to clarify values and preferences.

## Author Contributions

**David Forner**, study design, data collection, data analysis, manuscript preparation, manuscript approval; **Victoria Taylor**, data collection, data analysis, manuscript preparation, manuscript approval; **Martin Corsten**, study design, manuscript approval; **Valeria E. Rac**, data analysis, manuscript preparation, manuscript approval; **Sonia Meerai**, data analysis, manuscript preparation, manuscript approval; **Andrew G. Shuman**, study design, manuscript approval; **Sharon Tzelnick**, data collection, manuscript approval; **Rosemary Martino**, study design, manuscript approval; **John R. de Almeida**, study design, manuscript approval; **David P. Goldstein**, study design, data analysis, manuscript preparation.

## Disclosures

### Competing interests

None.

### Funding source

None.

## Supporting information

Supplemental Figure 1. Semi‐structured interview guide.
