# Supplementary material for: Needs Assessment for a Decision Aid in Oral Cancer Requiring Major Resection and Reconstructions
Source: Otolaryngol Head Neck Surg. 2025 Nov 18;174(1):120–7. doi: 10.1002/ohn.70071 (PMC12794741; doi:10.1002/ohn.70071)
Supplement: Supplementary file 1 — Supplemental Figure 1. Semi‐structured interview guide. [file OHN-174-120-s001.docx]

*Semi-Structured Interview Questions (Patients):*

1. [open] What are your thoughts about the information your surgeon gave you?
2. [open] What did you think about ***how*** that information was communicated to you?
3. [open] What do you think about when I say “sharing the decision to have surgery”
4. [open] Which information was the most important for you and why?
   Example given: The advantages, disadvantages of surgery, the duration of the recovery, the side effects and complications etcetera.
5. [open] How were your own personal values discussed?
6. [open] How were your preferences to have surgery or not to have surgery included in the discussion?
7. [open] How were you involved in making the decision to have surgery?
8. [open] What did you think about the decision-making process in general?
9. [open] What parts of the information your surgeon gave you were confusing?
10. [open] How would you improve the decision-making process?
11. [open] What would you have liked to know more about?
12. [open] What did you consider when making a decision to proceed with surgery?
13. [open] What was the role of your friends and family when making a decision?
    Example: family, friends, coworkers, etc
14. [open] How did you feel about making your decision?
    **Preamble regarding decision aids**: We are hoping to make a type of pamphlet or a website that helps both patients and doctors talk about the decision to have surgery or not. For some people this can be a difficult decision and it can be hard to talk about. We want to make sure this type of thing would be helpful for patients and so we are interested in hearing about what you think should be included.
15. [open] We call such things decision aids - What information would be important for you to have in that type of pamphlet? What information would help you make decisions regarding your treatment?

E.g what would you like to have known more about?

1. What format would you want such a pamphlet to be in? E.g print or online
2. [open] Do you have anything else to add about the decision-making process that we haven’t talked about?
3. [open] Do you have anything else to add about the decision aid that we haven’t talked about?
